# *catena*-Poly[[[di­aqua­bis­(seleno­cyanato-κ*N*)iron(II)]-μ-1,2-bis­(pyridin-4-yl)ethane-κ^2^ *N*:*N*′] 1,2-bis­(pyridin-4-yl)ethane disolvate dihydrate]

**DOI:** 10.1107/S1600536813012312

**Published:** 2013-05-15

**Authors:** Susanne Wöhlert, Inke Jess, Christian Näther

**Affiliations:** a Institut für Anorganische Chemie, Christian-Albrechts-Universität Kiel, Max-Eyth-Strasse 2, 24118 Kiel, Germany

## Abstract

The title compound, {[Fe(NCSe)_2_(C_12_H_12_N_2_)(H_2_O)_2_]·2C_12_H_12_N_2_·2H_2_O}_*n*_, was obtained by the reaction of iron(II) sulfate hepta­hydrate and potassium seleno­cyanate with 1,2-bis­(pyridin-4-yl)ethane (bpa) in water. The Fe^II^ cation is coordinated by two *N*-bonded seleno­cyanate anions, two water mol­ecules and two 1,2-bis­(pyridin-4-yl)ethane (bpa) ligands in a slightly distorted octa­hedral geometry. In addition, two non-coordinating bpa mol­ecules and two water mol­ecules are present. The Fe^II^ cation is located on a center of inversion while the coordinating bpa ligand is located on a twofold rotation axis. The Fe^II^ cations are linked by the bpa ligands into chains along the *b*-axis direction, which are further connected into layers perpedicular to the *c* axis by O—H⋯N and O—H⋯O hydrogen bonds to the non-coordin­ating bpa and the water mol­ecules. The crystal studied was twinned by pseudo-merohedry (180° rotation along c*; contribution of the minor twin component 3.7%).

## Related literature

For background to this work see: Boeckmann & Näther (2011 ▶); Wöhlert *et al.* (2012 ▶); Boeckmann *et al.* (2012 ▶).
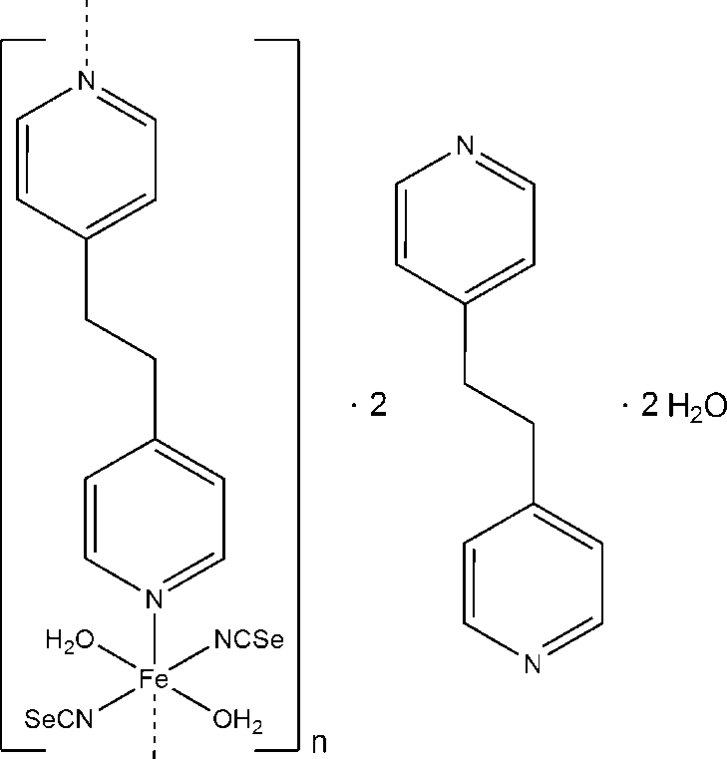


## Experimental

### 

#### Crystal data

[Fe(NCSe)_2_(C_12_H_12_N_2_)(H_2_O)_2_]·2C_12_H_12_N_2_·2H_2_O
*M* *_r_* = 890.58
Monoclinic, 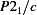

*a* = 8.0790 (6) Å
*b* = 14.1870 (7) Å
*c* = 17.6553 (12) Å
β = 102.645 (8)°
*V* = 1974.5 (2) Å^3^
*Z* = 2
Mo *K*α radiation
μ = 2.28 mm^−1^
*T* = 293 K
0.23 × 0.16 × 0.09 mm

#### Data collection

Stoe IPDS-2 diffractometer
Absorption correction: numerical (*X-SHAPE* and *X-RED32*; Stoe & Cie, 2008 ▶) *T*_min_ = 0.645, *T*_max_ = 0.818
20183 measured reflections
4540 independent reflections
3659 reflections with *I* > 2σ(*I*)
*R*_int_ = 0.095

#### Refinement

*R*[*F* ^2^ > 2σ(*F* ^2^)] = 0.045
*wR*(*F* ^2^) = 0.119
*S* = 1.09
4540 reflections
242 parameters
H-atom parameters constrained
Δρ_max_ = 0.58 e Å^−3^
Δρ_min_ = −0.88 e Å^−3^

### 

Data collection: *X-AREA* (Stoe & Cie, 2008 ▶); cell refinement: *X-AREA*; data reduction: *X-AREA*; program(s) used to solve structure: *SHELXS97* (Sheldrick, 2008 ▶); program(s) used to refine structure: *SHELXL97* (Sheldrick, 2008 ▶); molecular graphics: *XP* in *SHELXTL* (Sheldrick, 2008 ▶) and *DIAMOND* (Brandenburg, 2011 ▶); software used to prepare material for publication: *XCIF* in *SHELXTL* and *publCIF* (Westrip, 2010 ▶).

## Supplementary Material

Crystal structure: contains datablock(s) I, global. DOI: 10.1107/S1600536813012312/zl2548sup1.cif

Structure factors: contains datablock(s) I. DOI: 10.1107/S1600536813012312/zl2548Isup2.hkl

Additional supplementary materials:  crystallographic information; 3D view; checkCIF report

## Figures and Tables

**Table 1:** Selected bond lengths (Å)

Fe1—O1	2.069 (3)
Fe1—N1	2.132 (3)
Fe1—N10	2.347 (3)

**Table 2:** Hydrogen-bond geometry (Å, °)

*D*—H⋯*A*	*D*—H	H⋯*A*	*D*⋯*A*	*D*—H⋯*A*
O1—H1*O*1⋯O2	0.82	1.89	2.696 (4)	166
O1—H2*O*1⋯O2^i^	0.82	1.87	2.672 (4)	165
O2—H1*O*2⋯N20^ii^	0.82	1.98	2.688 (4)	144
O2—H2*O*2⋯N21^iii^	0.82	1.92	2.682 (4)	155

Symmetry codes: (i) 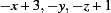; (ii) 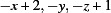; (iii) 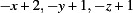.

**Fig. 1.**
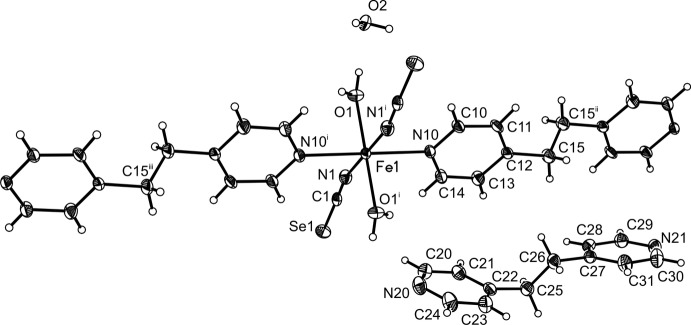
: Crystal structure of the title compound with labeling and displacement ellipsoids drawn at the 50% probability level. Symmetry code: i = -x + 2, -y, -z + 1; ii = -x + 2, -y + 1, -z + 1; iii = x, y - 1, z.

**Fig. 2.**
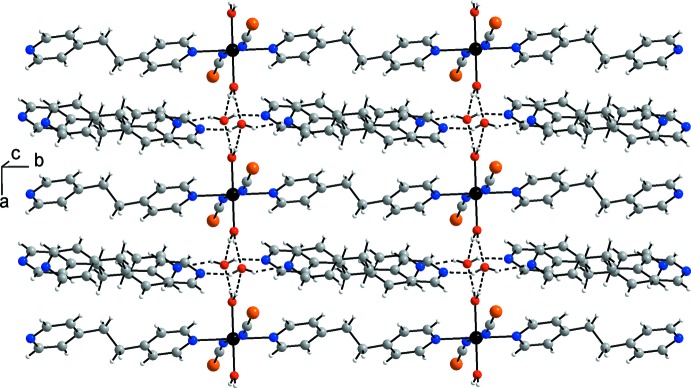
: Crystal structure of the title compound with O–H–N and O–H–O hydrogen bonding shown as dashed lines (black = iron, blue = nitrogen, orange = selenium, red = oxygen, grey = carbon, white = hydrogen).

## supplementary crystallographic information

### Comment

In our recent work we have reported on the synthesis and characterization of cobalt(II) and iron(II) selenocyanate coordination polymers that show coexistence of metamagnetism and a slow relaxation of the magnetization (Boeckmann & Näther, 2011; Wöhlert *et al.*, 2012 and Boeckmann *et al.*, 2012). These compounds can be prepared by thermal decomposition of suitable precursor compounds that contain volatile ligands like *e.g.* water in hydrates and therefore, such compounds are of extreme importance for our project. In the course of these investigations we obtained such a precursor based on 1,2-bis(pyridin-4-yl)ethane (bpa), which was characterized by single-crystal X-ray diffraction.

In the crystal structure of the title compound Fe(NCSe)_2_(C_12_H_12_N_2_)(H_2_O)_2_]*_n_*^.^2C_12_H_12_N_2_^.^2H_2_O solvate each Fe^II^ cation is coordinated by two *N*-bonded selenocyanate anions, two water molecules and two bpa ligands (Fig. 1). The FeN_4_O_2_ octahedra are slightly distorted with distances in the range of 2.071 (4) Å to 2.344 (4) Å. The angles arround the Fe^II^ cations are in the range of 86.91 (16) ° to 93.09 (16) ° and of 180 ° (Tab. 1). Each Fe^II^ cation is located on a center of inversion while the coordinating bpa ligand is located on a twofold rotation axis. The iron(II) cations are connected by the bpa ligands into one dimensional polymeric chains, which elongate in the direction of the crystallographic *b*-axis (Fig. 2). These chains are linked by intermolecular O—H···N and O—H···O hydrogen bonding into layers by non-coordinating bpa ligands and water molecules perpedicular to *c* (Fig. 2, Tab. 2).

### Experimental

FeSO_4_×7H_2_O, KNCSe and 1,2-bis(pyridin-4-yl)ethane were obtained from Alfa Aesar. All chemicals were used without further purification. 0.15 mmol (43 mg) FeSO_4_×7H_2_O and 0.2 mmol (28 mg) KNCSe were reacted with 0.6 mmol (109 mg) 1,2-bis(pyridin-4-yl)ethane in 1 ml water. Red single-crystals of the title compound were obtained after three days.

### Refinement

All H atoms were positions with idealized geometry and were refined isotropically with *U*_iso_(H) = 1.2 *U*_eq_(C) using a riding model with C—H = 0.93 Å and C—H_2_ = 0.97 Å. The O—H hydrogen atom were located in a difference map, their bond lengths were set to an ideal value of 0.82 Å, and finally they were refined using a riding model with *U*_iso_(H) = 1.5 *U*_eq_(O). Twinning by pseudo-merohedry of the crystal by a 180° rotation along *c** was taken into account, using a twin matrix (-1 0 0 0 -1 0 1 0 1; BASF parameter: 0.03696) which lowered the R value from 6.16 to 4.5%.

### Crystal data

**Table:**

[Fe(NCSe)_2_(C_12_H_12_N_2_)(H_2_O)_2_]·2C_12_H_12_N_2_·2H_2_O	*F*(000) = 908
*M**_r_* = 890.58	*D*_x_ = 1.498 Mg m^−^^3^
Monoclinic, *P*2_1_/*c*	Mo *K*α radiation, λ = 0.71073 Å
Hall symbol: -P 2ybc	Cell parameters from 20183 reflections
*a* = 8.0790 (6) Å	θ = 2.4–27.5°
*b* = 14.1870 (7) Å	µ = 2.28 mm^−^^1^
*c* = 17.6553 (12) Å	*T* = 293 K
β = 102.645 (8)°	Block, red
*V* = 1974.5 (2) Å^3^	0.23 × 0.16 × 0.09 mm
*Z* = 2	

### Data collection

**Table:**

Stoe IPDS-2 diffractometer	4540 independent reflections
Radiation source: fine-focus sealed tube	3659 reflections with *I* > 2σ(*I*)
Graphite monochromator	*R*_int_ = 0.095
ω scan	θ_max_ = 27.5°, θ_min_ = 2.4°
Absorption correction: numerical (*X-SHAPE* and *X-RED32*; Stoe & Cie, 2008)	*h* = −10→10
*T*_min_ = 0.645, *T*_max_ = 0.818	*k* = −18→18
20183 measured reflections	*l* = −22→22

### Refinement

**Table:**

Refinement on *F*^2^	Primary atom site location: structure-invariant direct methods
Least-squares matrix: full	Secondary atom site location: difference Fourier map
*R*[*F*^2^ > 2σ(*F*^2^)] = 0.045	Hydrogen site location: inferred from neighbouring sites
*wR*(*F*^2^) = 0.119	H-atom parameters constrained
*S* = 1.09	*w* = 1/[σ^2^(*F*_o_^2^) + (0.0393*P*)^2^ + 4.2316*P*] where *P* = (*F*_o_^2^ + 2*F*_c_^2^)/3
4540 reflections	(Δ/σ)_max_ = 0.001
242 parameters	Δρ_max_ = 0.58 e Å^−^^3^
0 restraints	Δρ_min_ = −0.88 e Å^−^^3^

### Special details

**Table:**

Geometry. All esds (except the esd in the dihedral angle between two l.s. planes) are estimated using the full covariance matrix. The cell esds are taken into account individually in the estimation of esds in distances, angles and torsion angles; correlations between esds in cell parameters are only used when they are defined by crystal symmetry. An approximate (isotropic) treatment of cell esds is used for estimating esds involving l.s. planes.
Refinement. Refinement of F^2^ against ALL reflections. The weighted R-factor wR and goodness of fit S are based on F^2^, conventional R-factors R are based on F, with F set to zero for negative F^2^. The threshold expression of F^2^ > 2sigma(F^2^) is used only for calculating R-factors(gt) etc. and is not relevant to the choice of reflections for refinement. R-factors based on F^2^ are statistically about twice as large as those based on F, and R- factors based on ALL data will be even larger.

### Fractional atomic coordinates and isotropic or equivalent isotropic displacement parameters (Å^2^)

**Table:**

	*x*	*y*	*z*	*U*_iso_*/*U*_eq_	
Fe1	1.0000	0.0000	0.5000	0.01501 (15)	
N1	1.0298 (4)	−0.0036 (2)	0.62294 (18)	0.0224 (6)	
C1	0.9954 (4)	−0.0081 (2)	0.6834 (2)	0.0174 (7)	
Se1	0.94592 (5)	−0.01632 (3)	0.77704 (2)	0.02554 (12)	
N10	0.9925 (4)	0.16528 (19)	0.50356 (18)	0.0189 (6)	
C10	1.0341 (5)	0.2181 (2)	0.4477 (2)	0.0229 (8)	
H10	1.0757	0.1878	0.4090	0.028*	
C11	1.0186 (5)	0.3159 (2)	0.4442 (2)	0.0246 (8)	
H11	1.0494	0.3489	0.4040	0.030*	
C12	0.9576 (5)	0.3639 (2)	0.5007 (2)	0.0223 (8)	
C13	0.9174 (6)	0.3093 (2)	0.5604 (2)	0.0251 (8)	
H13	0.8780	0.3379	0.6004	0.030*	
C14	0.9370 (5)	0.2119 (2)	0.5591 (2)	0.0229 (8)	
H14	0.9099	0.1770	0.5994	0.027*	
C15	0.9243 (6)	0.4693 (2)	0.4954 (3)	0.0353 (11)	
H15A	0.8506	0.4826	0.4454	0.042*	
H15B	0.8631	0.4864	0.5349	0.042*	
N20	0.5764 (5)	0.1671 (2)	0.6762 (2)	0.0319 (8)	
C20	0.6829 (6)	0.2156 (3)	0.7307 (3)	0.0319 (9)	
H20	0.7699	0.1830	0.7635	0.038*	
C21	0.6708 (6)	0.3120 (3)	0.7410 (3)	0.0296 (9)	
H21	0.7474	0.3429	0.7801	0.036*	
C22	0.5421 (5)	0.3616 (2)	0.6920 (2)	0.0254 (8)	
C23	0.4316 (6)	0.3110 (3)	0.6357 (3)	0.0320 (9)	
H23	0.3433	0.3414	0.6020	0.038*	
C24	0.4532 (6)	0.2150 (3)	0.6300 (3)	0.0342 (10)	
H24	0.3775	0.1821	0.5918	0.041*	
C25	0.5290 (6)	0.4669 (3)	0.6976 (2)	0.0291 (9)	
H25A	0.6028	0.4888	0.7453	0.035*	
H25B	0.4135	0.4844	0.6987	0.035*	
C26	0.5799 (6)	0.5132 (3)	0.6283 (2)	0.0277 (8)	
H26A	0.6899	0.4891	0.6241	0.033*	
H26B	0.4986	0.4955	0.5815	0.033*	
C27	0.5889 (5)	0.6193 (2)	0.6332 (2)	0.0229 (8)	
C28	0.6949 (5)	0.6639 (3)	0.6955 (2)	0.0253 (8)	
H28	0.7595	0.6288	0.7359	0.030*	
C29	0.7026 (5)	0.7616 (3)	0.6964 (2)	0.0283 (8)	
H29	0.7759	0.7909	0.7376	0.034*	
C30	0.5083 (7)	0.7723 (3)	0.5820 (3)	0.0365 (10)	
H30	0.4436	0.8091	0.5429	0.044*	
C31	0.4938 (6)	0.6753 (3)	0.5756 (3)	0.0329 (9)	
H31	0.4209	0.6480	0.5332	0.039*	
N21	0.6101 (5)	0.8156 (2)	0.6412 (2)	0.0309 (8)	
O1	1.2588 (3)	0.00213 (19)	0.50632 (15)	0.0241 (5)	
H1O1	1.2976	−0.0004	0.4672	0.036*	
H2O1	1.3430	−0.0061	0.5412	0.036*	
O2	1.4393 (4)	0.00140 (17)	0.39471 (15)	0.0227 (5)	
H1O2	1.3985	−0.0371	0.3612	0.034*	
H2O2	1.3942	0.0526	0.3828	0.034*	

### Atomic displacement parameters (Å^2^)

**Table:**

	*U*^11^	*U*^22^	*U*^33^	*U*^12^	*U*^13^	*U*^23^
Fe1	0.0173 (3)	0.0124 (3)	0.0164 (3)	0.0008 (2)	0.0059 (3)	0.0003 (2)
N1	0.0271 (16)	0.0203 (14)	0.0197 (15)	0.0008 (12)	0.0049 (13)	0.0006 (11)
C1	0.0183 (16)	0.0117 (14)	0.0204 (17)	0.0007 (12)	0.0007 (13)	−0.0002 (11)
Se1	0.0285 (2)	0.03071 (19)	0.01874 (19)	−0.00250 (16)	0.00811 (15)	−0.00011 (14)
N10	0.0242 (15)	0.0135 (13)	0.0199 (14)	0.0008 (11)	0.0067 (12)	0.0008 (10)
C10	0.032 (2)	0.0146 (15)	0.0268 (19)	−0.0005 (14)	0.0168 (17)	−0.0018 (13)
C11	0.034 (2)	0.0159 (15)	0.029 (2)	−0.0030 (14)	0.0198 (18)	0.0026 (13)
C12	0.0263 (19)	0.0118 (15)	0.032 (2)	−0.0015 (13)	0.0132 (17)	−0.0007 (13)
C13	0.037 (2)	0.0166 (16)	0.0266 (19)	0.0007 (15)	0.0166 (17)	−0.0031 (14)
C14	0.034 (2)	0.0151 (15)	0.0214 (17)	0.0023 (14)	0.0110 (16)	0.0017 (13)
C15	0.045 (3)	0.0125 (16)	0.059 (3)	0.0014 (16)	0.034 (2)	−0.0004 (16)
N20	0.044 (2)	0.0205 (15)	0.0312 (18)	0.0000 (14)	0.0075 (17)	−0.0019 (13)
C20	0.034 (2)	0.0256 (19)	0.036 (2)	0.0033 (16)	0.0073 (19)	0.0017 (16)
C21	0.033 (2)	0.0226 (18)	0.034 (2)	−0.0059 (15)	0.0077 (18)	−0.0015 (15)
C22	0.031 (2)	0.0181 (16)	0.032 (2)	−0.0014 (14)	0.0172 (18)	−0.0003 (14)
C23	0.038 (2)	0.0248 (19)	0.032 (2)	0.0006 (16)	0.0034 (19)	0.0013 (15)
C24	0.048 (3)	0.0226 (18)	0.029 (2)	−0.0054 (17)	0.003 (2)	−0.0039 (15)
C25	0.034 (2)	0.0204 (17)	0.037 (2)	−0.0012 (15)	0.0168 (19)	−0.0042 (15)
C26	0.037 (2)	0.0181 (16)	0.030 (2)	0.0010 (15)	0.0115 (18)	−0.0013 (14)
C27	0.0235 (19)	0.0179 (16)	0.029 (2)	0.0002 (13)	0.0098 (16)	−0.0015 (13)
C28	0.025 (2)	0.0213 (17)	0.032 (2)	0.0019 (14)	0.0101 (17)	0.0002 (14)
C29	0.031 (2)	0.0211 (17)	0.034 (2)	−0.0025 (15)	0.0099 (18)	−0.0030 (15)
C30	0.047 (3)	0.0242 (19)	0.037 (2)	0.0062 (18)	0.007 (2)	0.0079 (16)
C31	0.040 (2)	0.0262 (19)	0.030 (2)	−0.0027 (17)	0.0022 (19)	0.0004 (15)
N21	0.040 (2)	0.0194 (15)	0.036 (2)	0.0017 (14)	0.0137 (17)	0.0011 (13)
O1	0.0186 (13)	0.0350 (14)	0.0195 (12)	0.0013 (10)	0.0059 (11)	0.0015 (10)
O2	0.0271 (14)	0.0174 (11)	0.0231 (13)	0.0031 (10)	0.0043 (11)	−0.0012 (9)

### Geometric parameters (Å, º)

**Table:**

Fe1—O1	2.069 (3)	C21—H21	0.9300
Fe1—O1^i^	2.069 (3)	C22—C23	1.383 (6)
Fe1—N1^i^	2.132 (3)	C22—C25	1.503 (5)
Fe1—N1	2.132 (3)	C23—C24	1.379 (6)
Fe1—N10^i^	2.347 (3)	C23—H23	0.9300
Fe1—N10	2.347 (3)	C24—H24	0.9300
N1—C1	1.162 (5)	C25—C26	1.522 (6)
C1—Se1	1.788 (4)	C25—H25A	0.9700
N10—C10	1.339 (5)	C25—H25B	0.9700
N10—C14	1.339 (5)	C26—C27	1.508 (5)
C10—C11	1.393 (5)	C26—H26A	0.9700
C10—H10	0.9300	C26—H26B	0.9700
C11—C12	1.384 (5)	C27—C31	1.383 (6)
C11—H11	0.9300	C27—C28	1.390 (5)
C12—C13	1.402 (5)	C28—C29	1.388 (5)
C12—C15	1.519 (5)	C28—H28	0.9300
C13—C14	1.392 (5)	C29—N21	1.333 (5)
C13—H13	0.9300	C29—H29	0.9300
C14—H14	0.9300	C30—N21	1.330 (6)
C15—C15^ii^	1.481 (9)	C30—C31	1.384 (6)
C15—H15A	0.9700	C30—H30	0.9300
C15—H15B	0.9700	C31—H31	0.9300
N20—C24	1.327 (6)	O1—H1O1	0.8201
N20—C20	1.333 (6)	O1—H2O1	0.8201
C20—C21	1.386 (5)	O2—H1O2	0.8199
C20—H20	0.9300	O2—H2O2	0.8200
C21—C22	1.390 (6)		
			
O1—Fe1—O1^i^	180.000 (1)	C20—C21—C22	118.9 (4)
O1—Fe1—N1^i^	86.68 (12)	C20—C21—H21	120.5
O1^i^—Fe1—N1^i^	93.32 (12)	C22—C21—H21	120.5
O1—Fe1—N1	93.32 (12)	C23—C22—C21	117.5 (3)
O1^i^—Fe1—N1	86.68 (12)	C23—C22—C25	121.3 (4)
N1^i^—Fe1—N1	180.000 (1)	C21—C22—C25	121.1 (4)
O1—Fe1—N10^i^	89.09 (11)	C24—C23—C22	119.6 (4)
O1^i^—Fe1—N10^i^	90.91 (11)	C24—C23—H23	120.2
N1^i^—Fe1—N10^i^	89.69 (11)	C22—C23—H23	120.2
N1—Fe1—N10^i^	90.31 (11)	N20—C24—C23	123.4 (4)
O1—Fe1—N10	90.91 (11)	N20—C24—H24	118.3
O1^i^—Fe1—N10	89.09 (11)	C23—C24—H24	118.3
N1^i^—Fe1—N10	90.31 (11)	C22—C25—C26	109.9 (3)
N1—Fe1—N10	89.69 (11)	C22—C25—H25A	109.7
N10^i^—Fe1—N10	180.000 (1)	C26—C25—H25A	109.7
C1—N1—Fe1	160.1 (3)	C22—C25—H25B	109.7
N1—C1—Se1	178.9 (3)	C26—C25—H25B	109.7
C10—N10—C14	116.2 (3)	H25A—C25—H25B	108.2
C10—N10—Fe1	121.8 (2)	C27—C26—C25	113.8 (3)
C14—N10—Fe1	121.8 (2)	C27—C26—H26A	108.8
N10—C10—C11	123.7 (3)	C25—C26—H26A	108.8
N10—C10—H10	118.1	C27—C26—H26B	108.8
C11—C10—H10	118.1	C25—C26—H26B	108.8
C12—C11—C10	120.1 (3)	H26A—C26—H26B	107.7
C12—C11—H11	120.0	C31—C27—C28	117.9 (3)
C10—C11—H11	120.0	C31—C27—C26	121.3 (4)
C11—C12—C13	116.5 (3)	C28—C27—C26	120.8 (3)
C11—C12—C15	121.8 (3)	C29—C28—C27	118.8 (4)
C13—C12—C15	121.6 (3)	C29—C28—H28	120.6
C14—C13—C12	119.4 (3)	C27—C28—H28	120.6
C14—C13—H13	120.3	N21—C29—C28	123.3 (4)
C12—C13—H13	120.3	N21—C29—H29	118.3
N10—C14—C13	124.0 (3)	C28—C29—H29	118.3
N10—C14—H14	118.0	N21—C30—C31	123.3 (4)
C13—C14—H14	118.0	N21—C30—H30	118.3
C15^ii^—C15—C12	116.2 (5)	C31—C30—H30	118.3
C15^ii^—C15—H15A	108.2	C27—C31—C30	119.2 (4)
C12—C15—H15A	108.2	C27—C31—H31	120.4
C15^ii^—C15—H15B	108.2	C30—C31—H31	120.4
C12—C15—H15B	108.2	C30—N21—C29	117.4 (3)
H15A—C15—H15B	107.4	Fe1—O1—H1O1	121.5
C24—N20—C20	117.3 (3)	Fe1—O1—H2O1	134.5
N20—C20—C21	123.4 (4)	H1O1—O1—H2O1	102.6
N20—C20—H20	118.3	H1O2—O2—H2O2	108.6
C21—C20—H20	118.3		

Symmetry codes: (i) −*x*+2, −*y*, −*z*+1; (ii) −*x*+2, −*y*+1, −*z*+1.

### Hydrogen-bond geometry (Å, º)

**Table:**

*D*—H···*A*	*D*—H	H···*A*	*D*···*A*	*D*—H···*A*
O1—H1*O*1···O2	0.82	1.89	2.696 (4)	166
O1—H2*O*1···O2^iii^	0.82	1.87	2.672 (4)	165
O2—H1*O*2···N20^i^	0.82	1.98	2.688 (4)	144
O2—H2*O*2···N21^ii^	0.82	1.92	2.682 (4)	155

Symmetry codes: (i) −*x*+2, −*y*, −*z*+1; (ii) −*x*+2, −*y*+1, −*z*+1; (iii) −*x*+3, −*y*, −*z*+1.
